# Food groups, oils and butter, and cancer of the oral cavity and pharynx

**DOI:** 10.1038/sj.bjc.6690400

**Published:** 1999-05-01

**Authors:** S Franceschi, A Favero, E Conti, R Talamini, R Volpe, E Negri, L Barzan, C La Vecchia

**Affiliations:** Servizio di Epidemiologia, Centro di Riferimento Oncologico, Via Pedemontana Occ., 33081 Aviano (PN), Italy; Servizio di Epidemiologia (SIO), Istituto Regina Elena, Viale Regina Elena 291, 00161 Roma, Italy; Divisione di Anatomia Patologia, Centro di Riferimento Oncologico, Via Pedemontana Occ., 33081 Aviano (PN), Italy; Istituto di Ricerche Farmacologiche ‘Mario Negri’, Via Eritrea 62, 20157 Milano, Italy; Divisione di Otorinolaringoiatria, Azienda Ospedaliera 'S Maria degli Angeli’, Via Montereale, 33170 Pordenone, Italy; Istituto di Statistica Medica e Biometria, Universitá degli Studi di Milano, Via Venezian 1, 20133 Milano, Italy

**Keywords:** cancer of the oral cavity, cancer of the pharynx, diet, oil, butter

## Abstract

To elucidate the role of dietary habits, a study was carried out in 1992–1997 in the province of Pordenone in Northeastern Italy, and those of Rome and Latina in central Italy. Cases were 512 men and 86 women with cancer of the oral cavity and pharynx (lip, salivary glands and nasopharynx excluded) and controls were 1008 men and 483 women who had been admitted to local hospitals for a broad range of acute non-neoplastic conditions. The validated dietary section of the questionnaire included 78 foods or recipes and ten questions on fat intake patterns. After allowance for education, smoking, alcohol and total energy intake, significant trends of increasing risk with increasing intake emerged for soups, eggs, processed meats, cakes and desserts, and butter. Risk was approximately halved in the highest compared to the lowest intake quintile for coffee and tea, white bread, poultry, fish, raw and cooked vegetables, citrus fruit, and olive oil. The inverse association with oils, especially olive oil, was only slightly attenuated by allowance for vegetable intake. Thus, frequent consumption of vegetables, citrus fruit, fish and vegetable oils were the major features of a low-risk diet for cancer of the oral cavity and pharynx. © 1999 Cancer Research Campaign


*British Journal of Cancer* (1999) **80**(3/4), 614–620
© 1999 Cancer Research Campaign
Article no. bjoc.1998.0400

### Food groups, oils and butter, and cancer of the oral cavity and pharynx

**S Franceschi[1], A Favero[1], E Conti[2], R Talamini[1], R Volpe[3], E Negri[4], L Barzan[5] and C La Vecchia[4,6]**

[1]Servizio di Epidemiologia, Centro di Riferimento Oncologico, Via Pedemontana Occ., 33081 Aviano (PN), Italy; [2]Servizio di Epidemiologia (SIO), Istituto Regina Elena, Viale Regina Elena 291, 00161 Roma, Italy; [3]Divisione di Anatomia Patologia, Centro di Riferimento Oncologico, Via Pedemontana Occ., 33081 Aviano (PN), Italy; [4]Istituto di Ricerche Farmacologiche 'Mario Negri', Via Eritrea 62, 20157 Milano, Italy; [5]Divisione di Otorinolaringoiatria, Azienda Ospedaliera 'S Maria degli Angeli', Via Montereale, 33170 Pordenone, Italy; [6]Istituto di Statistica Medica e Biometria, Universitá degli Studi di Milano, Via Venezian 1, 20133 Milano, Italy

**Summary** To elucidate the role of dietary habits, a study was carried out in 1992–1997 in the province of Pordenone in Northeastern Italy, and those of Rome and Latina in central Italy. Cases were 512 men and 86 women with cancer of the oral cavity and pharynx (lip, salivary glands and nasopharynx excluded) and controls were 1008 men and 483 women who had been admitted to local hospitals for a broad range of acute non-neoplastic conditions. The validated dietary section of the questionnaire included 78 foods or recipes and ten questions on fat intake patterns. After allowance for education, smoking, alcohol and total energy intake, significant trends of increasing risk with increasing intake emerged for soups, eggs, processed meats, cakes and desserts, and butter. Risk was approximately halved in the highest compared to the lowest intake quintile for coffee and tea, white bread, poultry, fish, raw and cooked vegetables, citrus fruit, and olive oil. The inverse association with oils, especially olive oil, was only slightly attenuated by allowance for vegetable intake. Thus, frequent consumption of vegetables, citrus fruit, fish and vegetable oils were the major features of a low-risk diet for cancer of the oral cavity and pharynx.

**Keywords:** cancer of the oral cavity; cancer of the pharynx; diet; oil; butter

Cancers of the oral cavity and pharynx are together the fifth most common cancer in the world (Parkin et al, 1993). Although these tumours predominantly affect developing countries, steady increases in mortality have been observed in male cohorts born after 1910–1920, the largest increases having occurred in eastern and southern Europe (La Vecchia et al, 1998). More than 80% of cases of cancer of the oral cavity and pharynx in developed countries should be avoidable by elimination of tobacco smoking and heavy alcohol drinking (Negri et al, 1993). Correlations of these tumours with dietary habits are also, however, among the strongest ones observed for any site of malignancy, although fewer accurate data exist for cancer of the oral cavity and pharynx than, for instance, cancer of the colon-rectum or breast (World Cancer Research Fund, 1997).

Several case-control studies (Marshall et al, 1982; Winn et al, 1984; Notani and Jayant, 1987; McLaughlin et al, 1988; Franco et al, 1989; Rossing et al, 1989; Franceschi et al, 1991; La Vecchia et al, 1991; Oreggia et al, 1991; Gridley et al, 1992; Zheng et al, 1992; 1993; Levi et al, 1998) have consistently found that oral cancer patients have histories of diets low in fruit and vegetables, even after accounting for their high alcohol intake. Protective effects seemed strongest for citrus fruit and vegetables which are likely to be eaten uncooked (McLaughlin et al, 1988).

Increases in risk also seemed to derive from high intakes of foods that represented important sources of calories, such as starchy foods (Franceschi et al, 1991), pulses (Notani and Jayant, 1987), certain meats, especially processed meats, and eggs (Franceschi et al, 1991). These foods can be interpreted as markers of an unbalanced monotonous diet and vary from one place to another. The role of different types of fat on cancer of the oral cavity and pharynx has never been studied in detail (Lipworth et al, 1997), but data on squamous carcinomas of the hypopharynx (Estève et al, 1996) and the oesophagus (Tzonou et al, 1996) raised the possibility that saturated fat may exert an unfavourable influence, while certain vegetable oils be protective.

To further elucidate the role of different foods and dietary fats in cancer of the oral cavity and pharynx, we carried out a case-control study in two Italian areas. The use of a validated food frequency questionnaire allowed us to assess individual fat intake patterns and adjust findings for total energy intake, in addition to a number of non-dietary risk correlates. Furthermore, the large study size made it possible to study dietary correlates separately in four anatomic subsites.

### SUBJECTS AND METHODS

A case-control study of cancer of the oral cavity and pharynx was conducted between January 1992 and November 1997 in two Italian areas: the province of Pordenone in Northeastern Italy, and those of Rome and Latina in central Italy.

Cases had histologically confirmed cancer of the oral cavity and pharynx diagnosed no longer than 1 year prior to the interview and with no previous diagnoses of cancer at any site. Overall, 271 subjects with cancer of the oral cavity (219 men and 52 women, median age: 58, range 22–77 years) and 327 with cancer of the pharynx (293 men and 34 women, median age: 58, range 32–76 years) were included (Table 1).

*Received 24 August 1998*
*Accepted 21 October 1998*

*Correspondence to:* S Franceschi

**Table 1** Distribution of 271 cases of cancer of the oral cavity, 327 of the pharynx, and 1491 controls[a] according to selected characteristics. Italy, 1992–1997

| Characteristic | Cases | | Controls |
|---|---|---|---|
| | Oral cavity No. (%) | Pharynx No. (%) | No. (%) |
| Sex | | | |
| Male | 219 (81) | 293 (90) | 1008 (68) |
| Female | 52 (19) | 34 (10) | 483 (32) |
| Age group (years) | | | |
| < 40 | 14 (5) | 9 (3) | 115 (8) |
| 40–49 | 48 (18) | 62 (19) | 289 (19) |
| 50–59 | 89 (33) | 116 (35) | 461 (31) |
| 60–69 | 95 (35) | 116 (35) | 482 (32) |
| ≥ 70 | 25 (9) | 24 (7) | 144 (10) |
| Education (years) | | | |
| < 7 | 179 (67) | 231 (71) | 896 (60) |
| 7–11 | 57 (21) | 73 (23) | 400 (27) |
| ≥ 12 | 33 (12) | 20 (6) | 194 (13) |
| $\chi^2_{1\ \mathrm{Trend}}$ | 1.85 | 14.11 | |
| (*P*-value)[b] | (0.17) | (< 0.01) | |
| Smoking (cigarettes per day) | | | |
| Never | 35 (13) | 14 (4) | 585 (39) |
| < 15 | 36 (13) | 61 (19) | 174 (12) |
| 15–24 | 87 (32) | 112 (34) | 172 (12) |
| ≥ 25 (+ cigar and pipe) | 50 (18) | 50 (15) | 73 (5) |
| Ex smokers | 63 (23) | 90 (28) | 487 (33) |
| $\chi^2_{1\ \mathrm{Trend}}$ | 122.66 | 154.16 | |
| (*P*-value)[b,c] | (< 0.01) | (< 0.01) | |
| Alcohol intake (drinks per week) | | | |
| < 6.7 | 25 (9) | 17 (5) | 353 (24) |
| 6.8–15.7 | 20 (7) | 23 (7) | 400 (27) |
| 15.8–30.7 | 36 (13) | 43 (13) | 328 (22) |
| 30.8–57.7 | 63 (23) | 79 (24) | 286 (19) |
| ≥ 57.8 | 127 (47) | 165 (50) | 124 (8) |
| $\chi^2_{1\ \mathrm{Trend}}$ | 193.65 | 183.29 | |
| (*P*-value)[b] | (< 0.01) | (< 0.01) | |

[a]Some figures do not add up to the total because of some missing values. [b]Compared to the control group, adjusted for age, sex and centre. [c]Ex smokers excluded

The subsites were defined on the basis of the four-digit level of the International Classification of Diseases 9th Revision (WHO, 1977), i.e. tongue (ICD 9: 141.0–141.9) 163 cases; oral cavity (ICD 9: 143.0–145.8) 101 cases; oropharynx (ICD 9: 146.0–146.9) 199 cases; and hypopharynx (ICD 9: 148.0–148.9) 116 cases. Nineteen cases were attributed to unspecified parts of the oral cavity or pharynx (145.9 and 149) and were included in overall analyses, but not in subsite-specific ones. Cancer of the lip, salivary glands and nasopharynx were not considered.

Controls were patients with no history of cancer who were admitted to major hospitals in the same catchment areas as cases and who had acute, non-neoplastic conditions unrelated to smoking, alcohol drinking and long-term modifications of diet. They included 1008 men and 483 women (median age 57, range 20–78), belonging to the following diagnostic categories: traumas, mostly fractures and sprains (30%), other orthopaedic disorders, such as low back pain and disc disorders (24%); acute surgical conditions (20%); eye diseases (16%); and other miscellaneous diseases, such as skin and dental conditions (10%). In order to compensate for the rarity of cancer of the oral cavity and pharynx in women, a control-to-case ratio of greater then five was chosen for females, as opposed to two for males. On average, about 5% of cases and controls approached during hospital stay refused to be interviewed.

The same structured questionnaire and coding manual were used in each centre, and all interviewers were centrally trained and routinely supervised. Data checking for consistency and reliability were also conducted centrally. The questionnaire included information on socio-demographic characteristics, such as education and occupation, lifetime smoking and alcohol-drinking habits, physical activity, anthropometric measures at various ages, a problem-oriented personal medical history and family history of cancer.

### Food frequency questionnaire

The interviewer-administered food frequency questionnaire (FFQ) was developed to assess subjects' habitual diet, including total energy. Average weekly frequency of consumption of specific foods or food groups, as well as complex recipes (including the most common ones in the Italian diet) during the 2 years prior to cancer diagnosis or hospital admission (for controls) was elicited. Intakes lower than once a week, but at least once a month, were coded as 0.5 per week.

The FFQ included 78 foods, food groups or recipes. For 40 of the 78 foods, a serving size was indicated using natural units (e.g. one egg, one apple, etc.). For other foods, the possibility of specifying the use of a small, average or large portion was offered (Franceschi et al, 1993). Several questions aimed at assessing fat intake pattern (Franceschi et al, 1993). These addressed:

1. The type(s) of fat (i.e. olive oil; specific seed oils – safflower, maize, peanut and soya; mixed oils – including cheaper brands of variable composition; butter; and margarine) used as a condiment for raw and cooked vegetables, to prepare meat dishes, to fry, and to prepare pasta or rice dishes.
2. Subjective judgement on quantity of fat used (low, average, high).
3. Habits of eating the seasoning or sauce, or leaving it on the plate.

These questions, as well as frequency of consumption and portion size, were used to derive estimates of intake of various oils and fats. Satisfactory reproducibility (Franceschi et al, 1993, 1995) and validity (Decarli et al, 1996) of the FFQ have been reported.

### Statistical analysis

Food items and recipes were categorized into 19 food groups, i.e. milk (five questions), coffee and tea (four questions), white bread (three questions), pasta and rice dishes (six questions), soups (two questions), poultry (two questions), red meat (e.g. beef, veal, and pork; eight questions), processed meats (three questions), fish (three questions), cheese (five questions), pulses (one question), raw vegetables (four questions), cooked vegetables (six questions), potatoes (two questions), citrus fruit (two questions), other

© Cancer Research Campaign 1999

**Table 2** Odds ratios (OR) and corresponding 95% confidence intervals (CI)[a] according to intake quintile of food groups among 598 cases of cancer of the oral cavity and pharynx and 1491 controls. Italy, 1992–1997

| Food group Upper limit (servings per week) | Intake quintile 1[b] | 2 | 3 | 4 | 5 | $\chi^2_1$ Trend |
|---|---|---|---|---|---|---|
| Milk | 0.3 | 3.3 | 7.3 | 11.0 | – | |
| OR (95% CI) | 1 | 0.6 (0.4–0.9) | 0.7 (0.5–1.0) | 1.1 (0.8–1.7) | 1.0 (0.7–1.5) | 0.33 |
| Coffee and tea | 8.0 | 14.5 | 21.5 | 28.5 | – | |
| OR (95% CI) | 1 | 0.8 (0.5–1.1) | 0.9 (0.6–1.2) | 0.6 (0.4–0.9) | 0.6 (0.4–0.9) | 5.95[c] |
| White bread | 11.3 | 14.5 | 21.3 | 28.5 | – | |
| OR (95% CI) | 1 | 0.9 (0.7–1.4) | 0.7 (0.5–1.0) | 0.6 (0.4–0.9) | 0.4 (0.2–0.6) | 21.59[d] |
| Pasta and rice | 3.3 | 4.3 | 5.3 | 6.3 | – | |
| OR (95% CI) | 1 | 1.3 (0.9–1.9) | 0.9 (0.6–1.3) | 0.7 (0.5–1.0) | 1.1 (0.8–1.6) | 0.76 |
| Soups | 1.3 | 2.3 | 3.3 | 5.3 | – | |
| OR (95% CI) | 1 | 1.4 (1.0–2.0) | 1.4 (0.9–2.0) | 1.8 (1.2–2.6) | 2.5 (1.7–3.7) | 21.23[d] |
| Poultry | 1.0 | 1.5 | 2.5 | 3.5 | – | |
| OR (95% CI) | 1 | 0.9 (0.7–1.3) | 0.8 (0.5–1.1) | 0.6 (0.4–0.9) | 0.6 (0.4–0.9) | 10.04[d] |
| Red meat | 2.8 | 4.3 | 5.5 | 7.0 | – | |
| OR (95% CI) | 1 | 0.7 (0.5–1.0) | 1.1 (0.7–1.6) | 1.1 (0.7–1.6) | 1.2 (0.8–1.8) | 3.47 |
| Processed meats | 1.5 | 2.5 | 3.5 | 5.0 | – | |
| OR (95% CI) | 1 | 1.7 (1.2–2.3) | 1.7 (1.2–2.4) | 1.9 (1.2–2.8) | 1.6 (1.1–2.4) | 6.86[d] |
| Fish | 1.0 | 1.5 | 2.0 | 2.5 | – | |
| OR (95% CI) | 1 | 0.7 (0.5–1.0) | 0.7 (0.5–1.0) | 0.7 (0.5–1.0) | 0.6 (0.4–0.9) | 5.31[c] |
| Eggs | 1.0 | 2.0 | 2.5 | 4.0 | – | |
| OR (95% CI) | 1 | 1.5 (1.1–2.1) | 1.5 (1.1–2.1) | 2.0 (1.3–3.1) | 2.5 (1.7–3.7) | 23.13[d] |
| Cheese | 2.7 | 3.8 | 5.0 | 7.0 | – | |
| OR (95% CI) | 1 | 1.0 (0.7–1.4) | 0.9 (0.6–1.3) | 1.0 (0.7–1.5) | 1.2 (0.8–1.7) | 0.59 |
| Pulse | 0.5 | 1.0 | 2.0 | 3.0 | – | |
| OR (95% CI) | 1 | 1.6 (1.1–2.2) | 1.6 (1.1–2.3) | 1.1 (0.7–1.8) | 2.1 (1.0–4.2) | 2.48 |
| Raw vegetables | 5.0 | 8.0 | 10.5 | 14.1 | – | |
| OR (95% CI) | 1 | 0.4 (0.3–0.6) | 0.5 (0.3–0.7) | 0.5 (0.3–0.7) | 0.4 (0.3–0.6) | 13.83[d] |
| Cooked vegetables | 1.5 | 2.3 | 3.2 | 4.5 | – | |
| OR (95% CI) | 1 | 0.8 (0.5–1.1) | 1.0 (0.7–1.4) | 0.7 (0.5–1.0) | 0.5 (0.3–0.7) | 9.78[d] |
| Potatoes | 1.0 | 1.5 | 2.5 | 3.5 | – | |
| OR (95% CI) | 1 | 1.2 (0.8–1.8) | 1.3 (0.9–1.8) | 1.4 (0.9–2.0) | 1.1 (0.7–1.8) | 0.53 |
| Citrus fruit | 0.6 | 2.2 | 3.8 | 7.5 | – | |
| OR (95% CI) | 1 | 0.8 (0.5–1.1) | 0.7 (0.5–1.0) | 0.6 (0.4–0.8) | 0.5 (0.3–0.7) | 14.60[d] |
| Other fruit | 6.3 | 10.4 | 13.8 | 19.4 | – | |
| OR (95% CI) | 1 | 0.7 (0.5–1.0) | 0.7 (0.5–1.0) | 0.8 (0.6–1.2) | 0.7 (0.5–1.1) | 1.26 |
| Cakes and desserts | 0.7 | 2.0 | 4.1 | 8.3 | – | |
| OR (95% CI) | 1 | 1.0 (0.7–1.5) | 1.1 (0.7–1.5) | 1.0 (0.7–1.5) | 1.6 (1.1–2.4) | 4.24[c] |
| Sugar and candies | 14.5 | 27.5 | 40.5 | 58.0 | – | |
| OR (95% CI) | 1 | 1.0 (0.7–1.4) | 0.9 (0.6–1.3) | 0.8 (0.6–1.2) | 1.2 (0.8–1.7) | 0.10 |

[a]Estimates from multiple logistic regression equations including terms for age, centre, sex, education, smoking habit, total energy and alcohol intake. [b]Reference category. [c]$P < 0.05$. [d]$P < 0.01$

fruit (eight questions), cakes and desserts (seven questions), and refined sugar (including honey and candies; four questions).

The sum of the weekly servings of intake of food items and recipes included in the same group was approximately distributed into quintiles based on the entire study population. The intake of whole-grain bread, polenta (i.e. a maize porridge once common in Northeastern Italy; Franceschi et al, 1990), soft drinks and artificial sweeteners were assessed separately. With respect to vegetables and fruit, diversity was defined as the number of different types of vegetables and fruit eaten on average at least weekly.

Odds ratios (ORs), and the corresponding 95% confidence intervals (CIs) were computed, using unconditional multiple logistic regression models (Breslow and Day, 1980). All regression equations included terms for age (as a continuous variable), study centre, sex, years of education (< 7/7–11/≥ 12), smoking habits (never, former, < 20 and ≥ 20 cigarettes per day, current smoker, < 15, 15–24, and ≥ 25 cigarettes per day), alcohol drinking (as a continuous and categorical variable), and total energy intake (as a continuous variable). Since intake of various types of oils and fats tend to be inversely correlated, regression analyses included terms for all of them simultaneously and, when stated, total vegetable intake.

The intake frequency of each food group, and of olive oil and butter was also introduced as a continuous variable, and the unit of measurement for each food group was set at one serving per day. For sugar, one serving corresponded to four teaspoons; for olive oil to 30 g; and for butter to 10 g.

### RESULTS

Table 1 shows the distribution of cases of cancer of the oral cavity and pharynx and controls according to gender, age and selected characteristics. Females represented 19% of oral cancer cases, but only 10% of pharyngeal cancers. Cases of cancer of the pharynx, but not of oral cavity, reported significantly fewer educational

 *© Cancer Research Campaign 1999*

**Table 3** Odds ratios (OR) and corresponding 95% confidence intervals (CI)[a] among 598 cases of cancer of oral cavity and pharynx and 1491 controls according to intake quintile of oils and fats. Italy, 1992–1997

| Food group Upper limit (g per day) | Intake quintile | | | | | $X^2_1$ Trend |
|---|---|---|---|---|---|---|
| | 1[b] | 2 | 3 | 4 | 5 | |
| Olive oil | 3.2 | 18.3 | 29.5 | 42.9 | – | |
| OR (95% CI) | 1 | 0.6 (0.4–0.9) | 0.7 (0.5–1.1) | 0.7 (0.5–1.1) | 0.4 (0.3–0.7) | 7.15[c] |
| Vegetable-adjusted OR (95% CI) | 1 | 0.6 (0.4–0.9) | 0.8 (0.5–1.2) | 0.9 (0.5–1.4) | 0.6 (0.4–0.9) | 1.73 |
| Specific seed oils | 1.9 | 9.3 | – | | | |
| OR (95% CI) | 1 | 0.7 (0.5–1.0) | 0.6 (0.4–0.9) | | | 7.40[c] |
| Vegetable-adjusted OR (95% CI) | 1 | 0.7 (0.5–1.1) | 0.7 (0.4–1.0) | | | 4.88[d] |
| Mixed seed oils | 0.3 | 0.7 | 2.3 | 10.3 | – | |
| OR (95% CI) | 1 | 0.7 (0.5–1.1) | 1.0 (0.7–1.4) | 0.9 (0.6–1.3) | 1.1 (0.7–1.7) | 0.12 |
| Vegetable-adjusted OR (95% CI) | 1 | 0.7 (0.5–1.1) | 0.9 (0.6–1.4) | 0.9 (0.6–1.3) | 1.1 (0.7–1.7) | 0.15 |
| Butter | 0.4 | 0.9 | 1.9 | 7.7 | – | |
| OR (95% CI) | 1 | 1.2 (0.8–1.8) | 1.3 (0.8–1.9) | 1.8 (1.2–2.7) | 2.3 (1.6–3.5) | 22.32[c] |
| Vegetable-adjusted OR (95% CI) | 1 | 1.3 (0.9–1.9) | 1.3 (0.9–2.0) | 1.9 (1.3–2.7) | 2.4 (1.6–3.5) | 21.97[c] |
| Margarine | 0.2 | – | | | | |
| OR (95% CI) | 1 | 1.3 (0.8–2.2) | | | | 0.94 |
| Vegetable-adjusted OR (95% CI) | 1 | 1.3 (0.8–2.3) | | | | 0.98 |

[a]Estimates from multiple logistic regression equations including terms for age, centre, sex, education, smoking habit, total intake of alcohol and energy, plus all oils and fats above. [b]Reference category. [c]$P < 0.01$. [d]$P < 0.05$.

years than control subjects. For both cancer sites, a large excess of current smokers, especially heavy smokers, and heavy alcohol drinkers was found in cases as compared to controls.

Table 2 shows the upper limit of intake quintiles of major food groups and corresponding ORs. Intakes of milk, pasta and rice dishes, red meat, cheese, pulses, potatoes, fruit other than citrus fruit, and sugar and candies were not significantly related to cancer risk. A significant trend of increasing risk with increasing intake emerged for soups (OR in highest as compared to lowest quintile = 2.5, 95% CI: 1.7–3.7); eggs (OR = 2.5, 95% CI: 1.7–3.7); processed meats (OR = 1.6, 95% CI: 1.1–2.4); and cakes and desserts (1.6, 95% CI: 1.1–2.4). Seven food groups were inversely related to risk: coffee and tea (OR = 0.6; 95% CI: 0.4–0.9); white bread (OR = 0.4, 95% CI: 0.2–0.6); poultry (OR = 0.6, 95% CI: 0.4–0.9); fish (OR = 0.6, 95% CI: 0.4–0.9); raw vegetables (OR = 0.4, 95% CI: 0.3–0.6); cooked vegetables (OR = 0.5, 95% CI: 0.3–0.7); and citrus fruit (OR = 0.5, 95% CI: 0.3–0.7) (Table 2).

Whole-grain bread was consumed at least weekly by 5% of cases and 7% of controls (OR in highest as compared to lowest tertile 1.1; 95% CI: 0.6–1.9). As for polenta intake, a direct association emerged with an OR of 1.5 (95% CI: 1.1–2.1) in the highest compared to the lowest tertile. Daily intake of soft drinks was reported by 24% of cases and 14% of controls (OR in highest as compared to lowest tertile: 1.6, 95% CI: 1.3–2.2). Three percent of cases and 7% of controls reported use of artificial sweeteners (OR = 0.7, 95% CI: 0.4–1.2).

With respect to specific foods within groups, a positive association was found for both broth or light soups and vegetable soups. Among different types of processed meats, risk increases were seen for high intake of ham, salami and sausages, but not prosciutto (lean processed meat). All different types of raw vegetables, including carrots, tomatoes and mixed salads, exerted a significant favourable effect. Among cooked vegetables, marked protections emerged for artichokes and zucchini, eggplants and peppers, but not cruciferous vegetables. Beneficial effect from citrus fruit derived from both intake of the whole fruit and fresh citrus fruit juice. Among other fruit, significant inverse trends in risk were observed for peaches, apricots and prunes, melons, kiwi, and strawberries and cherries, but not apples and pears. A significant trend of increasing risk with the increase of intake was found for bananas and cooked fruit.

The influence of intake level of five types of oils or fats is evaluated in Table 3. High intake of olive oil (OR in highest vs lowest quintile = 0.4, 95% CI: 0.3–0.7) and specific seed oils (OR in highest vs lowest quintile = 0.6, 95% CI: 0.4–0.9) was associated with significantly lowered risk. The beneficial effect of olive oil and specific seed oils was attenuated by the introduction of vegetable intake (i.e. a variable directly associated with oil intake) in the model. Mixed seed oils and margarine were not significantly related to cancer risk whereas a strong positive association emerged for butter intake (OR = 2.3, 95% CI: 1.6–3.5).

All foods significantly related to cancer risk, including olive oil and butter, were entered simultaneously in a logistic regression equation, which included other dietary and non-dietary covariates, plus body mass index (data not shown). Associations shown in Tables 2 and 3 persisted, thus showing them to be independent from each other. Only the effects of poultry, processed meats, cakes and desserts, and olive oil were somewhat attenuated by mutual adjustment for other food groups.

ORs for an intake increase of one serving per day of each examined food group and olive oil and butter are shown in Table 4, separately for the two sexes, four subsites and overall. The influence of dietary habits was consistent in the two sexes. However, high intake of red meat was associated with a significant increase of cancer risk in women, but not in men, and the apparent protection from high intake of coffee and tea and citrus fruit was more marked in women. All findings were very similar for the four subsites. Overall, one more serving per day of food groups

*© Cancer Research Campaign 1999* 

**Table 4** Odds ratios and corresponding 95% confidence intervals[a] among 598 cases of cancer of the oral cavity and pharynx and 1491 controls for an intake increase of one daily serving of various foods, by sex, subsite, and overall. Italy, 1992–1997

| Food group | Odds ratio (95% confidence interval) | | | | | | |
|---|---|---|---|---|---|---|---|
| | Sex | | Subsite(s)[b] | | | | All |
| | Males | Females | Tongue | Oral cavity | Oropharynx | Hypopharynx | |
| Milk | 1.1 (1.0–1.3) | 1.0 (0.8–1.2) | 1.0 (0.8–1.2) | 0.9 (0.7–1.2) | 1.2 (1.0–1.4) | 1.1 (0.9–1.4) | 1.1 (1.0–1.2) |
| Coffee and tea | 1.0 (0.9–1.1) | 0.8 (0.7–1.0)[c] | 1.0 (0.9–1.1) | 0.9 (0.8–1.1) | 1.0 (0.9–1.1) | 0.9 (0.8–1.1) | 0.9 (0.9–1.0) |
| White bread | 0.8 (0.8–0.9) | 0.9 (0.7–1.1) | 0.8 (0.7–1.0) | 0.8 (0.6–0.9) | 0.8 (0.7–0.9) | 0.7 (0.6–0.9) | 0.8 (0.8–0.9) |
| Pasta and rice | 1.1 (0.7–1.7) | 0.6 (0.2–1.7) | 1.1 (0.5–2.1) | 1.1 (0.5–2.4) | 1.1 (0.6–2.2) | 1.0 (0.4–2.2) | 1.0 (0.6–1.5) |
| Soups | 2.6 (1.7–4.0) | 3.3 (1.5–7.1) | 2.0 (1.1–3.6) | 3.3 (1.7–6.5) | 3.2 (1.9–5.5) | 2.1 (1.0–4.3) | 2.7 (1.9–4.0) |
| Eggs | 2.6 (1.6–4.1) | 2.5 (0.7–8.5) | 1.8 (0.9–3.4) | 3.0 (1.5–5.9) | 3.3 (1.9–5.8) | 2.9 (1.5–5.9) | 2.5 (1.7–3.9) |
| Poultry | 0.4 (0.2–0.8) | 0.2 (0.0–1.3) | 0.5 (0.2–1.3) | 0.2 (0.1–0.9) | 0.4 (0.2–0.9) | 0.5 (0.2–1.5) | 0.4 (0.2–0.7) |
| Red meat | 1.2 (0.8–1.8) | 3.0 (1.2–7.2)[c] | 1.5 (0.9–2.4) | 1.0 (0.5–1.9) | 1.3 (0.8–2.2) | 1.5 (0.8–3.0) | 1.5 (1.0–2.1) |
| Processed meats | 1.4 (0.9–2.2) | 2.1 (0.8–5.4) | 1.7 (1.0–2.9) | 1.3 (0.7–2.4) | 1.0 (0.6–1.8) | 1.0 (0.5–1.9) | 1.5 (1.0–2.2) |
| Fish | 0.4 (0.1–1.0) | 0.6 (0.1–3.4) | 0.7 (0.2–2.5) | 0.4 (0.1–1.9) | 0.4 (0.1–1.6) | 0.2 (0.0–1.3) | 0.4 (0.2–1.0) |
| Cheese | 0.9 (0.6–1.2) | 1.4 (0.7–2.9) | 0.8 (0.5–1.3) | 0.8 (0.4–1.4) | 1.0 (0.6–1.5) | 0.9 (0.5–1.5) | 0.9 (0.7–1.3) |
| Pulse | 3.5 (1.0–12.9) | 1.4 (0.1–18.4) | 2.7 (0.5–14.5) | 2.7 (0.4–18.8) | 2.5 (0.4–15.2) | 0.6 (0.0–6.8) | 2.6 (0.8–8.1) |
| Raw vegetables | 0.7 (0.6–0.9) | 0.6 (0.4–0.9) | 0.7 (0.5–0.9) | 0.5 (0.4–0.8) | 0.7 (0.6–0.9) | 0.7 (0.5–1.0) | 0.7 (0.6–0.8) |
| Cooked vegetables | 0.6 (0.4–0.9) | 0.5 (0.2–1.3) | 0.5 (0.2–0.9) | 0.5 (0.2–1.1) | 0.5 (0.3–1.0) | 0.9 (0.4–1.8) | 0.6 (0.4–0.8) |
| Potatoes | 1.4 (0.7–2.9) | 0.7 (0.2–2.8) | 1.4 (0.6–3.5) | 4.0 (1.4–12.0) | 0.8 (0.3–1.9) | 1.3 (0.4–4.0) | 1.2 (0.7–2.3) |
| Citrus fruit | 0.8 (0.6–1.0) | 0.3 (0.2–0.6)[c] | 0.7 (0.5–1.0) | 0.5 (0.3–0.8) | 0.6 (0.4–0.9) | 0.9 (0.6–1.3) | 0.7 (0.5–0.9) |
| Other fruit | 1.0 (0.9–1.1) | 0.8 (0.7–1.0) | 0.9 (0.8–1.1) | 0.8 (0.7–1.0) | 0.9 (0.8–1.1) | 1.0 (0.8–1.2) | 0.9 (0.8–1.0) |
| Cakes and desserts | 1.2 (1.0–1.3) | 1.0 (0.8–1.4) | 1.0 (0.8–1.2) | 1.2 (1.0–1.5) | 1.2 (1.1–1.4) | 1.2 (1.0–1.5) | 1.1 (1.0–1.3) |
| Sugar and candies | 1.1 (1.0–1.2) | 1.2 (0.9–1.5) | 1.0 (0.8–1.2) | 1.1 (0.9–1.3) | 1.2 (1.0–1.4) | 1.2 (1.0–1.5) | 1.1 (1.0–1.2) |
| Olive oil | 0.7 (0.6–0.9) | 0.5 (0.3–0.9) | 0.7 (0.5–0.9) | 0.7 (0.5–1.1) | 0.5 (0.4–0.7) | 0.8 (0.5–1.1) | 0.7 (0.5–0.8) |
| Butter | 1.3 (1.1–1.6) | 1.6 (0.9–2.9) | 1.3 (1.0–1.8) | 1.4 (1.0–1.9) | 1.5 (1.1–1.9) | 1.2 (0.9–1.7) | 1.4 (1.1–1.6) |

[a]Estimates from multiple logistic regression equations including terms for age, sex, centre, education, smoking habit, alcohol and energy intake. [b]19 cancer cases whose subsite of origin was not specified are excluded. [c]$P < 0.05$ at Wald $\chi^2_1$ test for interaction.

associated with a favourable effect (i.e. poultry, fish, raw and cooked vegetables, citrus fruit, and olive oil) gave a risk reduction of 30% or over.

Separate analyses of dietary findings were performed by age (< 60 and ≥ 60 years of age), alcohol drinking (< 40 and ≥ 40 drinks per week) and smoking habits (never, light smokers and heavy smokers). No significant or consistent heterogeneity was found (data not shown).

Finally, risks by approximate tertile of diversity in the intake of vegetables and fruit are presented in Table 5. Consumption of many different types of vegetables (OR = 0.5, 95% CI: 0.3–0.8) and fruit (OR = 0.6, 95% CI: 0.4–0.9) seemed to exert a significant protection against cancer of the oral cavity and pharynx. Allowance for total number of servings of vegetables and fruit, however, attenuated the favourable effect of diversity, especially for fruit.

### DISCUSSION

Our study, one of the largest investigations on cancer of the oral cavity and pharynx, provides further support to the beneficial effect of high intake of most types of vegetables and fruit, and shows, for the first time, an inverse association with high intake of olive oil. Conversely, it suggests that cases had a diet significatively richer in soups, processed meats, eggs, cakes and desserts, and butter than control subjects. All major dietary findings were remarkably similar in different strata of sex and age and in four cancer subsites. Furthermore, despite strong direct associations with smoking and alcohol drinking, neither of these factors accounted for, or significantly modified, our dietary findings.

With the exception of the protective effect of high vegetable and fruit intake and related micronutrients, very little is known about dietary patterns associated with high risk of cancer of the oral

**Table 5** Odds ratios (OR) and corresponding 95% confidence intervals (CI) according to diversity of intake of food groups among 598 cases of cancer of oral cavity and pharynx and 1491 controls. Italy 1992–1997

| Food group Diversity[a] | OR (95% CI)[a,b] | OR (95% CI)[a,c] Adjusted for number of servings |
|---|---|---|
| *Vegetables* | | |
| < 4 | 1.0 | 1.0 |
| 4 – < 7 | 0.7 (0.5–0.9) | 0.8 (0.6–1.1) |
| ≥ 7 | 0.5 (0.3–0.8) | 0.6 (0.3–1.0) |
| $\chi^2_1$ trend | 14.28[d] | 4.34[e] |
| *Fruit* | | |
| < 4 | 1.0 | 1.0 |
| 4 – < 6 | 0.9 (0.7–1.1) | 1.0 (0.7–1.3) |
| ≥ 6 | 0.6 (0.4–0.9) | 0.7 (0.4–1.2) |
| $\chi^2_1$ trend | 4.91[e] | 1.12 |

[a]Types of vegetables and fruit consumed at least weekly. [b]Estimates from multiple logistic regression equations including terms for age, centre, education, sex, smoking habit, alcohol and energy intake. [c]Same as above, plus total weekly servings of vegetables and fruit. [d]$P < 0.01$. [e]$P < 0.05$.

© Cancer Research Campaign 1999

cavity and pharynx (World Cancer Research Fund, 1997). Not only are studies relatively few, but, with some exceptions (McLaughlin et al, 1988; Marshall et al, 1982, 1992), they have been relatively small and based on simplified dietary questionnaires which have not allowed an accurate assessment of fat intake patterns or adjustment for total energy intake.

Our study was carried out in a southern European population where: (1) the risk of cancer of the oral cavity and pharynx is relatively high (La Vecchia et al, 1998); (2) pharynx is over-represented in comparison with most published series (McLaughlin et al, 1988); and (3) broad ranges of intake of olive oil and alcoholic beverages exist.

With respect to vegetable and fruit intakes, virtually all studies that have examined vegetables as a broad category have reported inverse associations with risk (Notani and Jayant, 1987; Oreggia et al, 1991; Franceschi et al, 1992; Gridley et al, 1992; Levi et al, 1998). The highest intake frequency category showed, as in our study, an approximate halving of risk. The evidence is most consistent for carrots and raw vegetables (Franco et al, 1989; Franceschi et al, 1991, 1992; La Vecchia et al, 1991; Zheng et al, 1992), but weak for cruciferous vegetables and pulses, for which null or positive associations can be found (McLaughlin et al, 1988; Zheng et al, 1993), as in our study.

A protective effect of fruit, as a broad category, has also emerged consistently (Winn et al, 1984; McLaughlin et al, 1988; Franco et al, 1989; La Vecchia et al, 1991; Franceschi et al, 1992; Gridley et al, 1992; Zheng et al, 1992, 1993; Levi et al, 1998). In our study, high intakes of most types of fruit were associated with decreased risk, but only the effect of citrus fruits was significant. Thus, we did not confirm a more beneficial effect of fruits as compared to vegetables, including cooked vegetables (McLaughlin et al, 1988; Zheng et al, 1992). The combined benefit from high oil intake, which is regularly eaten with vegetables in Italy, may account for this discrepancy.

The disclosure of an important role of dietary fats is probably the most original finding of our study. Information on this issue was scanty. Notani and Jayant (1987) reported a beneficial effect of high intake of ground nut oil in a population from India where butter was very uncommon. In the largest study available, McLaughlin et al (1988) showed a direct association with saturated fat (OR = 1.5 in men and 1.3 in women in the highest intake quartile), but were not able to distinguish butter from margarine, not did they ask about vegetable oils (Gridley et al, 1992).

A significant association with butter intake was found in one previous study from northern Italy (Franceschi et al, 1991), but not in a second one (La Vecchia et al, 1991). In both, however, only a subjective score for dietary fat intake was available. Zheng et al (1993) and Chyou et al (1995) reported reduced risk (0.6 in both) in the highest quartile of total fat intake in two populations (Chinese in China, and Japanese in Hawaii) where saturated fat intake was low. Conversely, in the USA, Marshall et al (1992) reported a positive association with total fat intake after allowance for total energy.

Fat intake was assessed in detail in a multicentre European study on cancer of the hypopharynx and larynx (Estève et al, 1996). Results for cancer of the hypopharynx, which represents about 1/5 of the cases in our study, were very similar, with OR of 3.1 for the highest intake quintile of butter and 0.7 for oil, after allowance for alcohol, tobacco and energy intake. Similar findings emerged from two case-control studies of squamous cell carcinomas of the oesophagus in France (Launoy et al, 1998) and Greece (Tzonou et al, 1996). In terms of potential biologic mechanisms, olive oil may have endogenous antioxidant properties (Berry et al, 1995). It is not clear whether such antioxidant activity is due to oleic acid itself or to the presence of other antioxidants, such as vitamin E and polyphenols, in olive oil. Unexpectedly, fat intake patterns exerted a stronger influence on the risk of cancer of the oral cavity and pharynx than on colorectal cancer (Braga et al, 1998) or breast cancer (La Vecchia et al, 1995) in companion Italian studies.

Positive associations have been reported with the intake of certain types of meat (McLaughlin et al, 1988), especially pork products (Winn et al, 1984; Levi et al, 1998), nitrite-rich meat (McLaughlin et al, 1988), salted meat (Zheng et al, 1992; De Stefani et al, 1994) or charcoal-grilled meat (Franco et al, 1989), though generally not with fresh meat or poultry (La Vecchia et al, 1991). Similarly, we found an increased risk for a high intake of processed meat and eggs, although it is not clear whether such results should be attributed to high salt or nitrite content, or to high saturated fat and cholesterol (as suggested by the direct association with butter intake).

The interpretation of direct associations with the intake of soups and cakes and desserts is complex. Soups tend to be high in salt and may encompass frequent thermal injury if eaten hot (Chyou et al, 1995). Frequent intake of soups and cakes and desserts may be, however, correlates of poor oral hygiene and/or consequences of poor dentition in patients with cancer of the oral cavity and pharynx.

Coffee and tea, which were inversely associated with risk, may exert a mechanical cleansing effect in the oral cavity. A protective effect of high fish intake has already been reported (McLaughlin et al, 1988; Gridley et al, 1990), albeit not in investigations where salty fish predominated (Zheng et al, 1992; Chyou et al, 1995).

Finally, no direct association was found with pasta and rice such as has been observed in most previous work (McLaughlin et al, 1988; Chyou et al, 1995). High white bread intake showed an inverse relation to cancer risk, as demonstrated by Kjærheim et al (1998). A former report (Franceschi et al, 1990) of high risk of oral cavity and pharynx in heavy consumers of polenta (a porridge made from refined maize flour) was confirmed, although intake of this traditional food has been steadily declining.

Bias in the recall of food intake may well not be an important issue in a population such as the Italian one, which is generally not 'health conscious', and with hospital controls rather than 'healthy' non-hospital ones (Doll, 1994). The same may apply to selection bias, on account of much lower refusals to participate among hospital controls than non-hospital ones (Marshall et al, 1992). With respect to confounding, cancer of the oral cavity and pharynx showed a modest inverse association with educational level but, among study subjects, education was virtually uncorrelated with the intake of any of the food groups considered while the strongest risk determinants (i.e. smoking and drinking habits) were carefully allowed for.

In conclusion, the results of our study indicate that high intakes of vegetables, fruit, fish and vegetable oils, and low intake of soups, cakes, processed meats, eggs and butter were associated with reduced risk for both cancer of the oral cavity and pharynx in this Italian study population.

### ACKNOWLEDGEMENTS

This work was conducted within the contribution of the Italian Association for Research on Cancer. The authors wish to thank Dr

© Cancer Research Campaign 1999 

Maria Parpinel for her help with the food databases, Mrs Olinda Volpato for study coordination and Mrs A Redivo for editorial assistance.

### REFERENCES

Berry EM, Eisenberg S, Harats D, Friedlander Y, Norman N, Kaufmann NA and Stein Y (1995) Effects of diets rich in monounsaturated fatty acids on plasma lipoproteins – the Jerusalem Nutrition Study; monounsaturated vs saturated fatty acids. *Nutr Metab Cardiovasc Dis* **5**: 55–62

Braga C, La Vecchia C, Franceschi S, Negri E, Parpinel M, Decarli A, Giacosa A and Tricholoulos D (1998) Olive oil, other seasoning fats, and the risk of colorectal carcinoma. *Cancer* **82**: 448–453

Breslow NE and Day NE (1980) *Statistical Methods in Cancer Research. Vol. I, The Analysis of Case-control Studies*. International Agency for Research on Cancer, Scientific Publication no. 32. IARC: Lyon.

Chyou P-H, Nomura AMY and Stemmermann GN (1995) Diet, alcohol, smoking and cancer of the upper aerodigestive tract: a prospective study among Hawaii Japanese men. *Int J Cancer* **60**: 616–621

De Stefani E, Oreggia F, Ronco A, Fierro L and Rivero S (1994) Salted meat consumption as a risk factor for cancer of the oral cavity and pharynx: a case-control study from Uruguay. *Cancer Epidemiol Biomarkers Prev* **3**: 381–385

Decarli A, Franceschi S, Ferraroni M, Gnagnarella P, Parpinel MT, La Vecchia C, Negri E, Salvini S, Falcini F and Giacosa A (1996) Validation of a food-frequency questionnaire to assess dietary intakes in cancer studies in Italy. Results for specific nutrients. *Ann Epidemiol* **6**: 110–118

Doll R (1994) The use of meta-analysis in epidemiology: diet and cancers of the breast and colon. *Nutr Rev* **52**: 233–237

Estève J, Riboli E, Péquignot G, Terracini B, Merletti F, Crosignani P, Ascunce N, Zubiri L, Blanchet F, Raymond L, Repetto F and Tuyns AJ (1996) Diet and cancers of the larynx and hypopharynx: the IARC multi-center study in southwestern Europe. *Cancer Causes Control* **7**: 240–252

Franceschi S, Bidoli E, Barón AE and La Vecchia C (1990) Maize and risk of cancers of the oral cavity, pharynx, and esophagus in northeastern Italy. *J Natl Cancer Inst* **82**: 1407–1411

Franceschi S, Bidoli E, Barón AE, Barra S, Talamini R, Serraino D and La Vecchia C (1991) Nutrition and cancer of the oral cavity and pharynx in North East Italy. *Int J Cancer* **47**: 20–25

Franceschi S, Barra S, La Vecchia C, Bidoli E, Negri E and Talamini R (1992) Risk factors for cancer of the tongue and the mouth. *Cancer* **70**: 2227–2233

Franceschi S, Negri E, Salvini S, Decarli A, Ferraroni M, Filiberti R, Giacosa A, Talamini R, Nanni O, Panarello G and La Vecchia C (1993) Reproducibility of an Italian food frequency questionnaire for cancer studies: results for specific food items. *Eur J Cancer* **29A**: 2298–2305

Franceschi S, Barbone F, Negri E, Decarli A, Ferraroni M, Filiberti R, Giacosa A, Gnagnarella P, Nanni O, Salvini S and La Vecchia C (1995) Reproducibility of an Italian food frequency questionnaire for cancer studies. Results for specific nutrients. *Ann Epidemiol* **5**: 69–75

Franco EL, Kowalski LP, Oliveira BV, Curado MP, Pereira RN, Silva ME, Fava AS and Torloni M (1989) Risk factors for oral cancer in Brazil: a case-control study. *Int J Cancer* **43**: 992–1000

Gridley G, McLaughlin JK, Block G, Blot WJ, Gluch M and Fraumeni JF Jr (1992) Vitamin supplement use and reduced risk of oral and pharyngeal cancer. *Am J Epidemiol* **135**: 1083–1092

Gridley G, McLaughlin JK, Block G, Blot WJ, Winn DM, Greenberg RS, Schoenberg JB, Preston-Martin S, Austin DF and Fraumeni JF Jr (1990) Diet and oral and pharyngeal cancer among blacks. *Nutr Cancer* **14**: 219–225

Kjærheim K, Gaard M and Andersen A (1998) The role of alcohol, tobacco, and dietary factors in upper aerogastric tract cancers: a prospective study of 10 900 Norwegian men. *Cancer Causes Control* **9**: 99–108

Launoy G, Milan C, Day NE, Pienkowski MP, Gignoux M and Faivre J (1998) Diet and squamous-cell cancer of the oesophagus: a French multicentre case-control study. *Int J Cancer* **76**: 7–12

La Vecchia C, Negri E, D'Avanzo B, Boyle P and Franceschi S (1991) Dietary indicators of oral and pharyngeal cancer. *Int J Epidemiol* **20**: 39–44

La Vecchia C, Negri E, Franceschi S, Decarli A, Giacosa A and Lipworth L (1995) Olive oil, other dietary fats, and the risk of breast cancer (Italy). *Cancer Causes Control* **6**: 545–550

La Vecchia C, Negri E, Levi F, Decarli A and Boyle P (1998) Cancer mortality in Europe: effects of age, cohort of birth and period of death. *Eur J Cancer* **34**: 118–141

Levi F, Pasche C, La Vecchia C, Lucchini F, Franceschi S and Monnier P (1998) Food groups and risk of oral and pharyngeal cancer. *Int J Cancer* **77**: 705–709

Lipworth L, Martinez ME, Angell J, Hsieh C-C and Trichopoulos D (1997) Olive oil and human cancer: an assessment of the evidence. *Prev Med* **26**: 181–190

McLaughlin JK, Gridley G, Block G, Winn DM, Preston-Martin S, Schoenberg JB, Greenberg RS, Stemhagen A, Austin DF, Ershow AG, Blot WJ and Fraumeni JF Jr (1988) Dietary factors in oral and pharyngeal cancer. *J Natl Cancer Inst* **80**: 1237–1243

Marshall JR, Graham S, Mettlin C, Shedd D and Swanson M (1982) Diet in the epidemiology of oral cancer. *Nutr Cancer* **3**: 145–149

Marshall JR, Graham S, Haughey BP, Shedd D, O'Shea R, Brasure J, Wilkinson GS and West D (1992) Smoking, alcohol, dentition and diet in the epidemiology of oral cancer. *Eur J Cancer B Oral Oncol* **28B**: 9–15

Negri E, La Vecchia C, Franceschi S and Tavani A (1993) Attributable risk of oral cancer in northern Italy. *Cancer Epidemiol Biomarkers Prev* **2**: 189–193

Notani PN and Jayant K (1987) Role of diet in upper aerodigestive tract cancers. *Nutr Cancer* **10**: 103–113

Oreggia F, De Stefani E, Correa P and Fierro L (1991) Risk factors for cancer of the tongue in Uruguay. *Cancer* **67**: 180–183

Parkin DM, Pisani P and Ferlay J (1993) Estimates of the worldwide incidence of eighteen major cancers in 1985. *Int J Cancer* **54**: 594–606

Rossing MA, Vaughan TL and McKnight B (1989) Diet and pharyngeal cancer. *Int J Cancer* **44**: 593–597

Tzonou A, Lipworth L, Garidou A, Signorello LB, Lagiou P, Hsieh C-C and Trichopoulos D (1996) Diet and risk of esophageal cancer by histologic type in a low risk population. *Int J Cancer* **68**: 300–304

Winn DM, Ziegler RG, Pickle LW, Gridley G, Blot WJ and Hoover RN (1984) Diet in the etiology of oral and pharyngeal cancer among women from the southern United States. *Cancer Res* **44**: 1216–1222

World Cancer Research Fund in association with American Institute for Cancer Research (1997) *Food, Nutrition and the Prevention of Cancer: a Global Perspective*. World Cancer Research Fund and American Istitute for Cancer Research: Washington

World Health Organization (1997) *International Classification of Diseases, 9th Revision*. World Health Organization: Geneva

Zheng T, Boyle P, Willett WC, Hu H, Dan J, Evstifeeva TV, Niu S and MacMahon B (1993). A case-control study of oral cancer in Beijing, People's Republic of China. Associations with nutrient intakes, foods and food groups. *Eur J Cancer B Oral Oncol* **29B**: 45–55

Zheng W, Blot WJ, Shu X-O, Diamond EL, Gao Y-T, Ji B-T and Fraumeni JF Jr (1992) Risk factors for oral and pharyngeal cancer in Shanghai, with emphasis on diet. *Cancer Epidemiol Biomarkers Prev* **1**: 441–448

 © Cancer Research Campaign 1999
